# Intimate Partner Violence and Pregnancy and Infant Health Outcomes — Pregnancy Risk Assessment Monitoring System, Nine U.S. Jurisdictions, 2016–2022

**DOI:** 10.15585/mmwr.mm7348a1

**Published:** 2024-12-05

**Authors:** Megan Steele-Baser, Alyssa L. Brown, Denise V. D’Angelo, Kathleen C. Basile, Rosalyn D. Lee, Antoinette T. Nguyen, Cynthia H. Cassell

**Affiliations:** ^1^Division of Violence Prevention, National Center for Injury Prevention and Control, CDC; ^2^Division of Reproductive Health, National Center for Chronic Disease and Health Promotion, CDC; ^3^Oak Ridge Institute for Science and Education, Oak Ridge, Tennessee.

**Summary**

**What is already known about this topic?**

Intimate partner violence (IPV) during pregnancy is a preventable cause of injury and death with negative short- and long-term impacts for pregnant women, infants, and families.

**What is added by this report?**

During 2016–2022, among women with a live birth in nine jurisdictions, 5.4% experienced IPV during pregnancy. Emotional IPV (5.2%) was more common than physical (1.5%) and sexual (1.0%) IPV. All IPV types were associated with delayed or no prenatal care, depression and substance use during pregnancy, and low infant birth weight.

**What are the implications for public health practice?**

Addressing multiple IPV types through comprehensive prevention efforts is critical to supporting maternal and infant health.

## Abstract

Intimate partner violence (IPV) can include emotional, physical, or sexual violence. IPV during pregnancy is a preventable cause of injury and death with negative short- and long-term impacts for pregnant women, infants, and families. Using data from the 2016–2022 Pregnancy Risk Assessment Monitoring System in nine U.S. jurisdictions, CDC examined associations between IPV during pregnancy among women with a recent live birth and the following outcomes: prenatal care initiation, health conditions during pregnancy (gestational diabetes, pregnancy-related hypertension, and depression), substance use during pregnancy, and infant birth outcomes. Overall, 5.4% of women reported IPV during pregnancy. Emotional IPV was most prevalent (5.2%), followed by physical (1.5%) and sexual (1.0%) IPV. All types were associated with delayed or no prenatal care; depression during pregnancy; cigarette smoking, alcohol use, marijuana or illicit substance use during pregnancy; and having an infant with low birth weight. Physical, sexual, and any IPV were associated with having a preterm birth. Physical IPV was associated with pregnancy-related hypertension. Evidence-based prevention and intervention strategies that address multiple types of IPV are important for supporting healthy parents and families because they might reduce pregnancy complications, depression and substance use during pregnancy, and adverse infant outcomes.

## Introduction

Intimate partner violence (IPV) during pregnancy can cause maternal orthopedic and head injuries, obstetric complications, and fetal injury or death (*1*). Approximately 40% of homicides among persons known to be pregnant or within a year of pregnancy are related to IPV (*2*). IPV during pregnancy is also associated with delayed prenatal care (*3*), depression and posttraumatic stress disorder, substance use, and adverse birth outcomes (*4*). Some demographic groups experience a disproportionate prevalence of IPV during pregnancy, including Black or African American, American Indian or Alaska Native, multiracial, and younger women (*5*). Although studies have examined maternal mortality from violence, less is known about maternal morbidity from IPV (*6*). Similarly, the effects of emotional or sexual IPV during pregnancy are not as well understood as those resulting from physical violence (*3*,*6*). IPV can affect pregnancy health through physiologic responses to stress (*6*,*7*) and by influencing health-related behaviors, including use of prenatal care (*3*). This report examines associations between emotional, physical, sexual, or any IPV type during pregnancy and initiation of prenatal care, health conditions and substance use during pregnancy, and infant birth outcomes.

## Methods

### Data Source

The Pregnancy Risk Assessment Monitoring System (PRAMS) is a collaboration between CDC and 50 participating U.S. jurisdictions to conduct jurisdiction-specific, population-based surveillance on experiences before, during, and after pregnancy among women with a recent live birth. Participants are surveyed by mail or telephone 2–6 months postpartum (*8*). This report used 2016–2022 data from nine jurisdictions* that had data available for at least 1 study year and that included questions about emotional, physical, and sexual IPV during pregnancy. PRAMS data are weighted annually by jurisdiction to adjust for sample design, nonresponse, and noncoverage.^†^ The aggregate weighted data in this report represent the total population of women with a live birth in the included jurisdictions and years.

### Measures

Respondents were asked about experiencing emotional, physical, and sexual violence from their husband or partner during pregnancy^§^ (Supplementary Box, https://stacks.cdc.gov/view/cdc/170631). A dichotomous measure for any type of IPV was created by combining “yes” responses to one or more types of IPV-related experiences. Respondents also answered questions about initiation of prenatal care,^¶^ health conditions during pregnancy (gestational diabetes, pregnancy-related hypertension,** and depression), and substance use during pregnancy (cigarette smoking during the last 3 months of pregnancy, alcohol use during the last 3 months of pregnancy, and marijuana or illicit substance use any time during pregnancy).^††^ Information on infant birth outcomes, including low birth weight, preterm birth, small for gestational age, and large for gestational age, were obtained from linked birth certificate data.^§§^

### Data Analysis

The analysis includes 47,796 respondents from nine U.S. jurisdictions that collected information on emotional, physical, and sexual IPV during pregnancy. Weighted prevalence was estimated for each IPV type. Multivariable logistic regression was used to calculate adjusted prevalence ratios and 95% CIs for initiation of prenatal care, conditions during pregnancy, substance use during pregnancy, and birth outcomes by each IPV type experienced during pregnancy. Analyses for birth outcomes were restricted to singleton births.^¶¶^ All models were adjusted for respondent’s age, race and ethnicity, education, health insurance at delivery, and number of previous live births.*** In addition, models examining gestational diabetes, pregnancy-related hypertension, and birth outcomes were adjusted for prepregnancy body mass index.^†††^ These characteristics were selected a priori as potential confounders based on previous research (*1*,*3*–*6*). Statistical significance was determined by whether CIs overlapped the null of 1.0. Analyses were conducted using SAS-callable SUDAAN (version 11.0.4; RTI International) to account for complex survey design. This study was reviewed and approved by the Institutional Review Boards of CDC and participating jurisdictions.^§§§^

## Results

Overall, 5.4% of women reported any type of IPV during pregnancy: emotional (5.2%), physical (1.5%), and sexual (1.0%) (Figure). The adjusted prevalence of depression, cigarette smoking, and marijuana or illicit substance use during pregnancy among women who reported emotional, physical, sexual, or any IPV type during pregnancy was approximately twice that of women who did not report each respective IPV type (Table 1). Higher prevalences of delayed or no prenatal care and alcohol use during pregnancy were also found among women who reported emotional, physical, sexual, or any IPV type during pregnancy compared with women who did not report these IPV types. The prevalence of pregnancy-related hypertension among women who reported physical IPV during pregnancy was 1.30 times as high as that among those who did not, whereas the prevalence of gestational diabetes was 0.39 times lower than that among those not reporting physical IPV.

**FIGURE:**
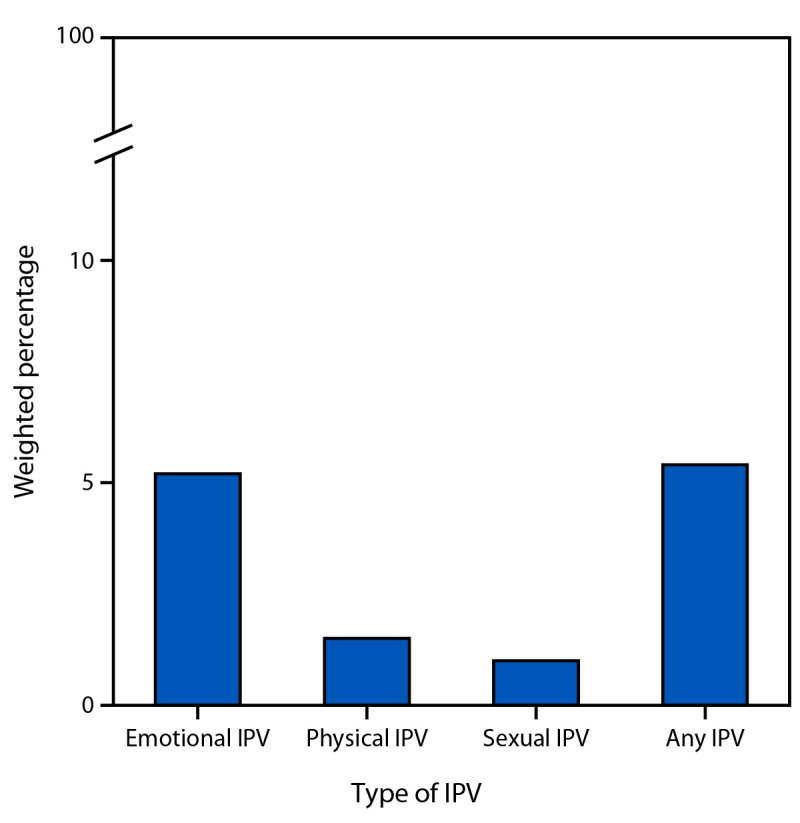
Prevalence of types of intimate partner violence during pregnancy among women with a recent live birth — Pregnancy Risk Assessment Monitoring System, nine U.S. jurisdictions, 2016–2022*^,^^†^ **Abbreviation:** IPV = intimate partner violence. * A total of 47,796 respondents were included in this analysis. Data from nine jurisdictions that met the response rate threshold for each year during the respective period (≥55% for 2016–2017 and ≥50% for 2018–2022): Arkansas (2016 and 2018–2021), District of Columbia (2018–2022), Indiana (2018), Kansas (2017–2022), Mississippi (2018–2021), Pennsylvania (2016–2022), South Dakota (2017–2022), Washington (2016–2022), and Wisconsin (2016–2022). ^†^ Each participating jurisdiction selects a monthly stratified sample of women from birth certificate records. Data were weighted by jurisdiction annually to adjust for sample design, noncoverage, and nonresponse and to represent the total population of women with a live birth in each jurisdiction in that year. The aggregate weighted data represents the total population of women with a live birth in the included jurisdictions and years.

**TABLE 1:** Association of types of intimate partner violence with prenatal care initiation, health conditions during pregnancy, and substance use among women with a recent live birth — Pregnancy Risk Assessment Monitoring System, nine U.S. jurisdictions, 2016–2022*

Type of IPV	Prenatal care^†^	Health conditions during pregnancy^†^			Substance use^†^		
	Delayed or no prenatal care^§^	Gestational diabetes^¶^	Pregnancy-related hypertension**	Depression^††^	Cigarette smoking during the last 3 months of pregnancy^§§^	Any alcohol use during the last 3 months of pregnancy^¶¶^	Marijuana or illicit substance use during pregnancy***
	aPR^†††^ (95% CI)	aPR^§§§^ (95% CI)	aPR^§§§^ (95% CI)	aPR^†††^ (95% CI)	aPR^†††^ (95% CI)	aPR^†††^ (95% CI)	aPR^†††^ (95% CI)
**Emotional IPV during pregnancy^¶¶¶^**							
Yes	1.38 (1.21–1.58)****	0.91 (0.72–1.15)	1.09 (0.94–1.26)	2.72 (2.50–2.96)****	2.32 (2.04–2.63)****	1.50 (1.15–1.97)****	2.71 (2.18–3.38)****
No	Ref	Ref	Ref	Ref	Ref	Ref	Ref
**Physical IPV during pregnancy^††††^**							
Yes	1.59 (1.30–1.94)****	0.61 (0.43–0.88)****	1.30 (1.03–1.64)****	2.57 (2.25–2.95)****	2.45 (2.01–3.00)****	1.96 (1.28–3.00)****	2.71 (1.95–3.75)****
No	Ref	Ref	Ref	Ref	Ref	Ref	Ref
**Sexual IPV during pregnancy^§§§§^**							
Yes	1.60 (1.24–2.07)****	0.90 (0.57–1.41)	0.79 (0.54–1.14)	2.67 (2.24–3.17)****	2.00 (1.51–2.64)****	1.99 (1.14–3.46)****	2.85 (1.94–4.18)****
No	Ref	Ref	Ref	Ref	Ref	Ref	Ref
**Any type of IPV during pregnancy^¶¶¶¶^**							
**Yes**	1.43 (1.26–1.62)****	**0.90 (0.72–1.13)**	**1.11 (0.97–1.28)**	2.71 (2.50–2.95)****	2.34 (2.07–2.65)****	1.48 (1.14–1.93)****	2.86 (2.31–3.54)****
**No**	**Ref**	**Ref**	**Ref**	**Ref**	**Ref**	**Ref**	**Ref**

**Abbreviations:** aPR = adjusted prevalence ratio; IPV = intimate partner violence; Ref = referent group.

* Data from nine jurisdictions that met the response rate threshold for each year during the respective period (≥55% for 2016–2017 and ≥50% for 2018–2022): Arkansas (2016 and 2018–2021), District of Columbia (2018–2022), Indiana (2018), Kansas (2017–2022), Mississippi (2018–2021), Pennsylvania (2016–2022), South Dakota (2017–2022), Washington (2016–2022), and Wisconsin (2016–2022).

^†^ All analyses were restricted to respondents with complete data on the respective outcome and demographic covariates: delayed or no prenatal care (45,446); gestational diabetes (44,251); pregnancy-related hypertension (44,179); depression during pregnancy (45,936); cigarette smoking during the last 3 months of pregnancy (46,281); any alcohol use during the last 3 months of pregnancy (25,189); and marijuana or illicit drug use during pregnancy (22,516). For alcohol use during pregnancy, the sample was restricted to respondents in four jurisdictions with available data: Mississippi, Pennsylvania, South Dakota, and Washington. For marijuana or illicit drug use during pregnancy, the sample was restricted to respondents in five jurisdictions with available data: District of Columbia, Indiana, Kansas, South Dakota, and Wisconsin.

^§^ Entry into prenatal care after first trimester or no prenatal care.

^¶^ Self-report of having gestational diabetes that started during their most recent pregnancy.

** Self-report of having high blood pressure that started during their most recent pregnancy or having preeclampsia or eclampsia during their most recent pregnancy.

^††^ Self-report of having depression during their most recent pregnancy.

^§§^ Smoked any cigarettes on an average day during the last 3 months of pregnancy.

^¶¶^ Had any alcoholic drinks in an average week during the last 3 months of pregnancy.

*** Used any of the following substances during their most recent pregnancy: marijuana or hash, heroin, amphetamines, cocaine, tranquilizers, or hallucinogens.

^†††^ Adjusted for respondent age, race and ethnicity, education, health insurance at delivery, and number of previous live births.

^§§§^ Adjusted for respondent age, race and ethnicity, education, health insurance at delivery, number of previous live births, and prepregnancy body mass index.

^¶¶¶^ Defined as husband or partner doing any of the following things to the respondent: threatened or made them feel unsafe in some way, made them frightened for their safety or their family’s safety because of the partner’s anger or threats, or tried to control their daily activities (e.g., controlling who they could talk to or where they could go).

**** Statistically significant; 95% CIs do not cross the null of 1.0.

^††††^ Defined as being pushed, hit, slapped, kicked, choked, or physically hurt in any way by their husband or partner.

^§§§§^ Defined as being forced to take part in touching or any sexual activity by their husband or partner when they did not want to.

^¶¶¶¶^ Any combination of physical, emotional, or sexual IPV by their husband or partner.

The prevalence of having an infant with low birth weight was higher among women who reported emotional, physical, sexual, or any IPV type than among those who did not report these IPV types (Table 2). The prevalence of having a preterm birth was higher among women who reported physical, sexual, or any IPV type than it was among those who did not report these IPV types.

**TABLE 2:** Association of types of intimate partner violence during pregnancy with infant birth outcomes among women with a recent live birth — Pregnancy Risk Assessment Monitoring System, nine U.S. jurisdictions, 2016–2022*

Type of IPV	Infant birth outcomes^†^			
	Low birth weight^§^	Preterm birth^¶^	Small for gestational age**	Large for gestational age^††^
	aPR^§§^ (95% CI)	aPR^§§^ (95% CI)	aPR^§§^ (95% CI)	aPR^§§^ (95% CI)
**Emotional IPV during pregnancy** ^¶¶^				
Yes	1.30 (1.11–1.51)***	1.18 (1.00–1.41)	1.04 (0.87–1.26)	1.04 (0.83–1.31)
No	Ref	Ref	Ref	Ref
**Physical IPV during pregnancy** ^†††^				
Yes	1.32 (1.03–1.69)***	1.50 (1.13–1.98)***	1.08 (0.79–1.49)	1.07 (0.74–1.55)
No	Ref	Ref	Ref	Ref
**Sexual IPV during pregnancy** ^§§§^				
Yes	1.47 (1.02–2.12)***	1.54 (1.07–2.21)***	1.10 (0.73–1.67)	1.16 (0.74–1.82)
No	Ref	Ref	Ref	Ref
**Any type of IPV during pregnancy** ^¶¶¶^				
**Yes**	1.29 (1.11–1.50)***	1.24 (1.05–1.47)***	**1.06 (0.88–1.26)**	**1.05 (0.84–1.30)**
**No**	**Ref**	**Ref**	**Ref**	**Ref**

**Abbreviations:** aPR = adjusted prevalence ratio; IPV = intimate partner violence; Ref = referent group.

* Data from nine jurisdictions that met the response rate threshold for each year during the respective period (≥55% for 2016–2017 and ≥50% for 2018–2022): Arkansas (2016 and 2018–2021), District of Columbia (2018–2022), Indiana (2018), Kansas (2017–2022), Mississippi (2018–2021), Pennsylvania (2016–2022), South Dakota (2017–2022), Washington (2016–2022), and Wisconsin (2016–2022).

^†^ All analyses were restricted to respondents with singleton births and complete data on the respective outcome and demographic covariates: low birth weight (42,832); preterm birth (42,873); small for gestational age (42,755); and large for gestational age (42,755).

^§^ Infant birth weight of <5.5 lbs (<2,500 g).

^¶^ Birth occurred at <37 weeks’ gestation.

** Infant birth weight <10th percentile for gestational age.

^††^ Infant birth weight >90th percentile for gestational age.

^§§^ Adjusted for respondent age, race and ethnicity, education, health insurance at delivery, number of previous live births, and prepregnancy body mass index.

^¶¶^ Defined as husband or partner doing any of the following things to the respondent: threatened or made them feel unsafe in some way, made them frightened for their safety or their family’s safety because of the partner’s anger or threats, or tried to control their daily activities (e.g., controlling who they could talk to or where they could go).

*** Statistically significant; 95% CIs do not cross the null of 1.0.

^†††^ Defined as being pushed, hit, slapped, kicked, choked, or physically hurt in any way by their husband or partner.

^§§§^ Defined as being forced to take part in touching or any sexual activity by their husband or partner when they did not want to.

^¶¶¶^ Any combination of physical, emotional, or sexual IPV by their husband or partner.

## Discussion

This analysis found that 5.4% of women with a live birth in nine U.S. jurisdictions during 2016–2022 experienced IPV while pregnant; emotional IPV was more commonly reported than physical and sexual IPV. Estimates of IPV during pregnancy vary across previous studies and might not include or differentiate between IPV types (*4*). Although studies have shown associations between maternal and infant outcomes and physical or combined IPV measures, research demonstrating their associations with emotional and sexual IPV is limited (*1**,**3**–**6*). This study found that all IPV types were associated with delayed or no prenatal care, depression and substance use during pregnancy, and infant low birth weight. Sexual, physical, and any IPV type also were associated with preterm birth. Depression and substance use during pregnancy can have cascading effects for the infant and family after birth.^¶¶¶^^,^**** Mental health-related deaths, including deaths by manner of suicide, overdose or poisoning related to substance use disorder, and other deaths determined to be related to a mental health condition, are the leading cause of pregnancy-related deaths.^††††^

In this analysis, physical IPV was associated with pregnancy-related hypertension, a risk factor for low birth weight, preterm birth, and stroke.^§§§§^ Physical IPV was also associated with a lower prevalence of gestational diabetes. The limited research evaluating this relationship has found no association or a higher prevalence (*7*); additional research is needed to clarify this association.

### Limitations

The findings in this report are subject to at least five limitations. First, findings can only be generalized to women with live births during 2016–2022 in the U.S. jurisdictions included in this report; for some indicators, such as alcohol use during pregnancy, only a subset of included jurisdictions collected this information. Findings for birth outcomes can only be generalized to women with a live singleton birth. Second, information regarding experiences during pregnancy is self-reported in the postpartum period and subject to recall and social desirability biases (e.g., respondents might underreport sensitive experiences such as IPV or substance use). Third, IPV estimates were based on respondents’ reports of acts by a husband or partner and did not include acts by ex-partners. Thus, findings likely underestimated the prevalence of IPV during pregnancy. Fourth, the IPV measures do not reflect the frequency or severity of violence or combinations of IPV types, all of which could affect the strength of associations between IPV and outcomes. Finally, reference groups for IPV measures differed by the type of IPV examined, with each reference group representing the absence of that specific IPV type. As a result, associations between different IPV types and outcomes cannot be directly compared. 

### Implications for Public Health Practice

This report reinforces the importance of recognizing emotional, physical, and sexual IPV during pregnancy as a serious public health concern. Prevention strategies work best when they operate across the social-ecological model, addressing factors at personal, relationship, community, and societal levels (*9*). Primary prevention strategies such as teaching healthy relationship skills and strengthening economic support for families might reduce IPV (*9*). Screening and referral by health care providers can also connect patients with services. This report highlights the need to assess multiple IPV types and provide interventions and resources. The U.S. Preventive Services Task Force recommends that health care providers screen women of reproductive age for IPV and refer those with indication of IPV to ongoing support services (*10*). IPV screening is a covered preventive service provided at no cost to patients.^¶¶¶¶^ Universal prevention education and information about community resources (e.g., mental health services, crisis hotlines, and shelters) can be provided to all persons regardless of IPV disclosure (*9*). Because pregnant women experiencing IPV are less likely to receive timely prenatal care, prevention education and intervention in other program models, such as home visitation programs, can be considered (*9*). By implementing comprehensive screening and intervention measures, combined with evidence-based primary prevention strategies (*9*), experiences of IPV could be reduced. Increasing awareness about the negative impacts of IPV during pregnancy and implementing effective strategies are critical for promoting the health of pregnant women, infants, and families.

## Footnotes

* Data from nine jurisdictions that met the response rate threshold for each year during the respective period (≥55% for 2016–2017 and ≥50% for 2018–2022): Arkansas (2016 and 2018–2021), District of Columbia (2018–2022), Indiana (2018), Kansas (2017–2022), Mississippi (2018–2021), Pennsylvania (2016–2022), South Dakota (2017–2022), Washington (2016–2022), and Wisconsin (2016–2022).

† Each participating jurisdiction selects a monthly stratified sample of women from birth certificate records. Data were weighted by jurisdiction annually to adjust for sample design, noncoverage, and nonresponse and to represent the total population of women with a live birth in each jurisdiction in that year. PRAMS aggregate data are not weighted to provide national estimates. https://www.cdc.gov/prams/php/methodology/index.html

§ Questions for emotional and sexual IPV during pregnancy asked only about violence by a husband or partner. Although the question for physical IPV during pregnancy has a response option for “ex-husband or ex-partner,” only physical IPV by a husband or partner was included to align with emotional and sexual IPV questions. https://www.cdc.gov/prams/php/questionnaires/

¶ Prenatal care initiation was based on respondent self-report of how many weeks or months pregnant they were at their first prenatal care visit. Delayed or no prenatal care was defined as entry into prenatal care after the first trimester or report of no prenatal care.

** Pregnancy-related hypertension was based on respondent self-report of having high blood pressure that started during their most recent pregnancy or having preeclampsia or eclampsia during their most recent pregnancy.

†† Data on alcohol use during pregnancy were available from four jurisdictions: Mississippi, Pennsylvania, South Dakota, and Washington. Data on marijuana or illicit drug use during pregnancy were available from five jurisdictions: District of Columbia, Indiana, Kansas, South Dakota, and Wisconsin.

§§ Low birth weight was defined as a birth weight of <5.5 lbs (<2,500 g), preterm birth was defined as birth at a gestational age of <37 weeks, small for gestational age was defined as a birth weight <10th percentile for gestational age, and large for gestational age was defined as a birth weight >90th percentile for gestational age.

¶¶ For infant birth outcomes, analyses were restricted to singleton births because measures were based on singleton growth and birth weight references.

*** Information on respondent age, race and ethnicity, education, health insurance at delivery, and number of previous live births was obtained from linked birth certificate data.

††† Prepregnancy body mass index was calculated from prepregnancy height and weight self-reported on the PRAMS questionnaire. https://www.cdc.gov/bmi/about/index.html

§§§ 45 C.F.R. part 46, 21 C.F.R. part 56.

¶¶¶
https://www.cdc.gov/maternal-infant-health/pregnancy-substance-abuse/index.html

****
https://www.aap.org/en/patient-care/perinatal-mental-health-and-social-support/

††††
https://www.cdc.gov/maternal-mortality/php/data-research/index.html

§§§§
https://www.cdc.gov/maternal-infant-health/pregnancy-complications/

¶¶¶¶
https://www.hrsa.gov/womens-guidelines
